# Dietary Adherence Is Associated with Perceived Stress, Anhedonia, and Food Insecurity Independent of Adiposity

**DOI:** 10.3390/nu16040526

**Published:** 2024-02-14

**Authors:** Jetaun M. Booker, Tomás Cabeza de Baca, Andrés M. Treviño-Alvarez, Emma J. Stinson, Susanne B. Votruba, Douglas C. Chang, Scott G. Engel, Jonathan Krakoff, Marci E. Gluck

**Affiliations:** 1 Obesity and Diabetes Clinical Research Section, Phoenix Epidemiology and Clinical Research Branch, National Institute of Diabetes and Digestive and Kidney Diseases, National Institutes of Health, Phoenix, AZ 85016, USA; jetaun.booker@gmail.com (J.M.B.); andres.trevino-alvarez@nih.gov (A.M.T.-A.); emma.stinson@nih.gov (E.J.S.); votrubas@niddk.nih.gov (S.B.V.); douglas.chang2@nih.gov (D.C.C.); jkrakoff@mail.nih.gov (J.K.); gmarci@mail.nih.gov (M.E.G.); 2 Sanford Research, Fargo, ND 58103, USA; scott.engel@sanfordhealth.org

**Keywords:** dietary adherence, perceived stress, food insecurity, anhedonia

## Abstract

We examined whether perceived stress, anhedonia, and food insecurity were associated with dietary adherence during a 6-week intervention. Sixty participants (23 m; 53 ± 14 y) completed psychosocial measures and were provided with full meals. Individuals with obesity were randomized to a weight-maintaining energy needs (WMENs) (*n* = 18; BMI 33 ± 4) or a 35% calorie-reduced diet (n = 19; BMI 38 ± 9); normal-weight individuals (n = 23; BMI 23 ± 2) were assigned to a WMENs diet. Adherence scores were determined via weekly assessments and daily ecological momentary assessments (EMAs) of real-time behavior in a natural environment. Perceived stress and anhedonia were associated with % body fat (all r-values > 0.25, all *p*-values < 0.05), but food insecurity and adherence were not. Higher perceived stress (*r* = −0.31, *p* = 0.02), anhedonia (*r* = −0.34, *p* = 0.01), and food insecurity (*r* = −0.27, *p* = 0.04) were associated with lower adherence scores, even after adjusting for age, sex, and % body fat. In all adjusted models, % body fat was not associated with adherence. Higher measures of stress, anhedonia, and food insecurity predicted lower adherence independently of body fat, indicating that psychosocial factors are important targets for successful adherence to dietary interventions, regardless of body size.

## 1. Introduction

Adherence is defined as “the extent to which patients follow the instructions that are given to them for prescribed treatments” [1] and plays a significant role in successful health outcomes. Medication adherence improves patient outcomes [2,3,4] and dietary adherence is a predictor for successful weight loss [5,6,7,8]. However, the factors that underlie adherence are not well understood [9].

We previously conducted a study examining the role of adiposity on dietary adherence. Contrary to our hypothesis, we observed that normal-weight individuals and individuals with obesity did not differ in dietary adherence, and that adherence to a weight-maintaining or calorie-reduced diet was not associated with perceived hunger [10]. 

In a secondary analysis, we demonstrated that adherence measured in a free-living environment by ecological momentary assessment (EMA) was predicted by trait-level affect and food insecurity [11]. Moreover, food insecurity moderated the relationship between low adherence and negative trait-level affect [11] such that food insecurity was associated with maladaptive eating behaviors, objective overeating, and higher binge eating scores [12].

The literature, including several of our studies, has shown that other psychosocial factors like perceived stress and anhedonia also affect adherence [9,13,14,15] and, along with food insecurity, are associated with obesity, metabolic syndrome, increased energy intake, poor diet [16,17,18,19,20,21], and weight gain [12,22,23]. Previous theories have outlined multiple psychosocial stress mechanisms that might have an impact on the risk of obesity [24], specifically concerning eating patterns and self-regulatory behavior. We found that at six-month follow-up, perceived stress predicted weight gain (*r*^2^ = 0.23, *p* = 0.02) and higher anhedonia scores predicted weight gain (*r*^2^ = 0.24, *p* = 0.04) at 1 year [22]. 

However, no studies, to our knowledge, have examined the interactive or combined effects of stress, anhedonia, and food insecurity. Moreover, existing studies exploring the influence of broadly defined psychosocial factors on adherence to dietary interventions have largely focused on subjects without obesity and, to our knowledge, none have included those following weight-maintaining diets. Therefore, the aim of the following analysis was to examine the role of psychosocial factors (perceived stress, anhedonia, and food insecurity) on adherence in adults with and without obesity and in those following a weight-maintaining or under-feeding diet [10]. We hypothesized that higher perceived stress, higher anhedonia, and higher food insecurity would predict a lower adherence to the dietary intervention. 

## 2. Materials and Methods

This was a secondary analysis of a clinical trial (ClinicalTrials.gov identifier: NCT01862796) examining dietary adherence [10]. Individuals aged 18 to 70 years were recruited from the greater Phoenix, Arizona, area between May 2013 and March 2018. They were invited to participate in a 6-week outpatient dietary intervention program. Weight-stable (±2% for past 3 months) participants with BMI < 25 kg/m^2^ (normal weight) and ≥30 kg/m^2^ (those with obesity) who were on no medications and otherwise healthy based on physical examination and laboratory tests, which included a full chemistry panel (e.g., complete blood cell count, serum creatinine, ALT, AST, GGT, TSH, fasting plasma glucose, and HbA1c), a urine sample for urinalysis, drug screen, nicotine test, and pregnancy test (if female), were recruited. Potential participants were excluded if they had a BMI ≥ 26 kg/m^2^ and BMI ≤ 29 kg/m^2^, significant health problems, current or past 3-month use of prescribed medication that might affect weight, smoking history, or excess alcohol (>3 drinks/d), substance abuse or dependence, or were in treatment for obesity or receiving psychotherapy. Of those screened (n = 100), 61 were eligible. Participants with obesity (BMI ≥ 30 kg/m^2^) were randomized to either a 35% calorie-reduced (CR) diet or a weight-maintaining energy needs (WMENs) diet. All the individuals in the normal-weight group (BMI < 25 kg/m^2^) were on a WMENs diet. For analyses, one participant in the normal-weight group was excluded because he started taking an excluded medication during the study period. Screening, eligibility, and analyzed data are reported in the CONSORT diagram (Supplemental Figure S1). Participants in the current analysis were from the same cohort as those included in the primary analysis that was previously published by Stinson et al., 2019 [10]. All participants were informed of the nature, purpose, and risks of the study. They all provided written informed consent prior to participation. The study protocol [13DKN096] was approved by the Institutional Review Board of the National Institute of Diabetes and Digestive and Kidney Diseases, and complied with the guidelines of the Helsinki Convention.

### 2.1. Study Design

#### 2.1.1. Baseline Visits

Participants completed a battery of psychological questionnaires and cognitive performance tests during their first baseline visit. They also underwent dual-energy X-ray absorptiometry (DXA; GE Healthcare Lunar iDXA and Lunar Prodigy) to determine body composition; DXA outputs were standardized using a reference prediction equation [25]. A weight-maintaining energy needs (WMENs) diet was calculated for each participant based on their specific weight, height, and sex, as previously reported [26], and Physical Activity Recall questionnaire [27,28]. Participants also met with a study counselor during this time to discuss their 4-day WMENs diet prescription. Of their daily caloric intake, 20% was provided as protein, 30% as fat, and 50% as carbohydrates. Their foods were prepackaged meals and snacks provided by our metabolic kitchen. They were given 4 days of food and were instructed to eat only the food we provided for them, to not eat any additional foods, to maintain their current levels of physical activity, and to continuously update their self-monitoring food record form. During the same week, they came back to the unit for a second baseline visit. Their weight was measured and they were provided with an additional 3-day supply of food. The WMENs diet was adjusted by 200 kcal if their weight exhibited a change of ±2%.

#### 2.1.2. Outpatient Visits (6 Weeks)

During the first outpatient visit, participants with obesity were stratified by sex and age using a block design. An investigator who was not part of the study randomized them to receive either a 35% CR diet or to continue their WMENs diet. All normal-weight participants continued their WMENs diet. Participants were given 4 days of food and the same dietary and study instructions they received during their baseline visit. Additionally, they were taught how to use momentary data collection via a smartphone system (described below). Participants visited the unit twice per week during which time they were weighed and picked up their meals. During their first visit of the week, participants met with a study counselor to collect food records.

#### 2.1.3. Adherence Assessments at Weekly In-Person Visits

Also, during the first visit each week, participants met with a second member of the study staff (to avoid bias) and completed a 24-h food recall. They also completed a similar 24-h recall on a computer in private. The computerized survey included additional questions assessing hunger levels (How hungry do you feel right now?) and the liking of the food (How much do you enjoy the taste of the study food that has been provided to you?) on a 5-point Likert scale (1 = low, 5 = high).

#### 2.1.4. Outpatient Adherence Assessments

During the 6-week period, adherence was assessed in two additional ways:

1. A 24-h food recall was conducted once a week. A member of the study staff called participants at random times via phone.
2. Ecological momentary assessment (EMA) was obtained twice daily using a smartphone data collection system called ReTAINE (https://retaine.org/, accessed on 9 February 2024). Participants were signaled once between 8 AM and 3 PM and once between 3 PM and 9 PM to assess behavior in their natural environment. Participants were asked, “*Since the last time you were signaled, have you eaten anything?*”, “*If yes, did you eat the study food provided to you?*”, “*If no, which food didn’t you eat?*”, “*Did you eat anything else (in addition to the food provided)?*”, and “*If yes, what did you eat?*”.

### 2.2. Study Predictors

The following self-reported psychosocial measures were all collected during the baseline visit. The instruments were administered by pencil and paper by trained staff members who were supervised by a clinical psychologist (MEG).

#### 2.2.1. Perceived Stress

Perceived stress was measured using the validated Perceived Stress Scale (PSS; [29]), which included 14 items and assessed stress in the last 28 days via 4 domains as follows: unpredictability, lack of control, burden overload, and stressful life circumstances. An example item is “*In the last month, how often have you been able to control irritations in your life?*”. Answer choices included ‘Never’, ‘Almost never’, ‘Sometimes’, ‘Fairly often’, and ‘Very often’. There were 6 positively stated items: 4, 5, 6, 7, 9, 10, 13. These items were reverse-scored, then all 14 items were summed to produce a total score. A higher score denoted higher perceived stress. This measure has high internal consistency (between 0.84 and 0.86) and strong validity [30,31,32,33].

#### 2.2.2. Anhedonia

The Physical Anhedonia Scale (PAS; [34]) is a 61-item measure that assesses sensitivity to reward. In otherwise healthy individuals, anhedonia as measured using the PAS tool assesses anhedonic traits rather than symptoms specific to a condition (e.g., major depressive disorder). An example item is “*When I pass by flowers, I have often stopped to smell them*”. The response choices were ‘True’ or ‘False’. A score of 1 was given to responses answered in the deviant (anhedonic) direction and a score of ‘0’ for responses answered in the hedonic direction. For example, if the participant answered ‘True’ to the above item, they received a ‘0’ because they answered in the hedonic (enhanced ability to seek out and enjoy natural rewards) direction. However, if they answered ‘False’ in the anhedonic direction, they received a ‘1’. Therefore, high scores on the scale indicated high levels of anhedonia, e.g., low ability to seek out and enjoy natural rewards. The Physical Anhedonia Scale has high internal consistency (mid to high 80s) and good validity [34,35,36].

#### 2.2.3. Food Insecurity

Participants completed the validated USDA Household Food Security Short Form (FSQ; [37]). Levels of food security were measured using 6 items centered around the participant’s ability to afford nutritionally rich foods. An example item from the questionnaire is “*In the last 12 months, were you ever hungry but didn’t eat because there wasn’t enough money for food?*”. Responses included, ‘Yes’, ‘No’, and ‘Don’t know’. A continuous score was created by summing the responses of all the items. A higher score indicated less food security. Reliability assessments of the FSQ have demonstrated that the measure is very reliable, with previously reported alphas residing in the mid to high 0.80 range [38,39]. Prior validity assessments have also demonstrated that the measure performs as expected, predicting applicable appetitive outcomes [37].

#### 2.2.4. Subjective Social Status

The MacArthur Scale of Subjective Social Status [40] is a ladder diagram with ten rungs. Participants were asked to place themselves on the rung that represented their community-level status compared to others (e.g., “*Where would you place yourself on this ladder, compared to others in your community?*”). Higher rungs represented greater subjective social status and the value was treated as a continuous score. Statistical assessments of reliability cannot be obtained on 1-item measures, but responses from the subjective social status measure demonstrates ample evidence of convergent and divergent validity (see [41,42] for reviews).

### 2.3. Scoring Adherence

Adherence was coded as a binary variable: 0 points awarded if nonadherent and 1 point awarded if adherent. There were 6 assessments of adherence. Participants were awarded points through (1) food diaries, (2) 24-h recall in-person interview, (3) computer survey, (4) 24-h outpatient telephone recall, (5) attendance at both weekly appointments, and (6) being on time 9 (±15 min) for the once-a-week counselor appointments. Adherence points were awarded for the first four measures for (1) completing the assessment, (2) eating all the food provided, and (3) not eating any additional foods. Participants could earn up to 72 adherence points for 6 weeks from these four assessments. Participants could also earn up to 18 possible points for attending both weekly appointments and being on time for the counselor sessions. Over the course of the study, participants could earn up to 90 possible points from these six assessments.

#### 2.3.1. EMA Recordings

Twice a day, participants were signaled via their smartphones to complete the EMA. Adherence points were awarded for (1) completing the EMA, (2) eating all the food provided, and (3) not eating additional foods. A daily average score was obtained by dividing the EMA weekly scores by 7. With six points possible per day, participants were able to earn 36 total possible points over the course of the study.

#### 2.3.2. Total Adherence Score

Based on prior principal components analysis on our adherence measures [10], a total adherence score was calculated for each participant by summing all the points they received from each measure of adherence during the 6-week period [10]. Total percent adherence score was then derived by dividing each participant’s total adherence score by the total possible points available. A thorough description and example of the scoring algorithm can be found in the work by Stinson et al. [10].

### 2.4. Covariates

Multivariable analyses included the following covariates: age (in years), sex (male, female), percent body fat (%), and community-level assessment of subjective social status. 

### 2.5. Statistical Analysis

Analyses were performed using SAS 9.4 (SAS Institute, Inc., Cary, NC, USA). Descriptive statistics were presented as mean (M) and standard deviation (*SD*) for continuous variables and counts (n) and percentages (%) for categorical variables. Descriptive statistics were stratified by randomized dietary group status (normal weight and individuals with obesity (WMENs and CR)). The two groups with obesity were combined into one group to strengthen the statistical power of the analysis. No significant differences were found between the two groups with obesity on adherence or with the psychosocial questionnaires [10]. Pearson correlation coefficients (*r*) were computed to assess the unadjusted intercorrelations between the predictors, covariates, and the outcome of interest (see Supplemental Table S1 for complete Pearson and Spearman coefficients’ correlation matrix). Separate general linear models were calculated to examine the association between psychosocial factors (perceived stress, anhedonia, and food insecurity) and adherence, accounting for age, sex, and percent body fat (Model 1) and age, sex, percent body fat, and subjective social studies (Model 2). Statistical significance was denoted as *p* < 0.05 (two-tailed).

## 3. Results

Sixty participants (23 male; age 53.3 ± 14.5 years; normal weight n = 23; BMI 22.7 ± 1.8, those with obesity n = 37; BMI 35.8 ± 7.2) had data available for analysis (Table 1). Participants with obesity scored significantly higher for perceived stress compared to normal-weight participants (B = 4.54, *p* = 0.03, CI = 0.57, 8.52), but not after adjusting for the covariates. No significant differences were observed between normal-weight individuals and individuals with obesity on physical anhedonia or food insecurity.

### 3.1. Pearson Correlation Analyses

Greater perceived stress was associated with higher anhedonia scores (*r* = 0.46, *p* = 0.0002) and greater food insecurity (*r* = 0.36, *p* = 0.0053), but there was no significant association between anhedonia and food insecurity.

**Figure 1:**
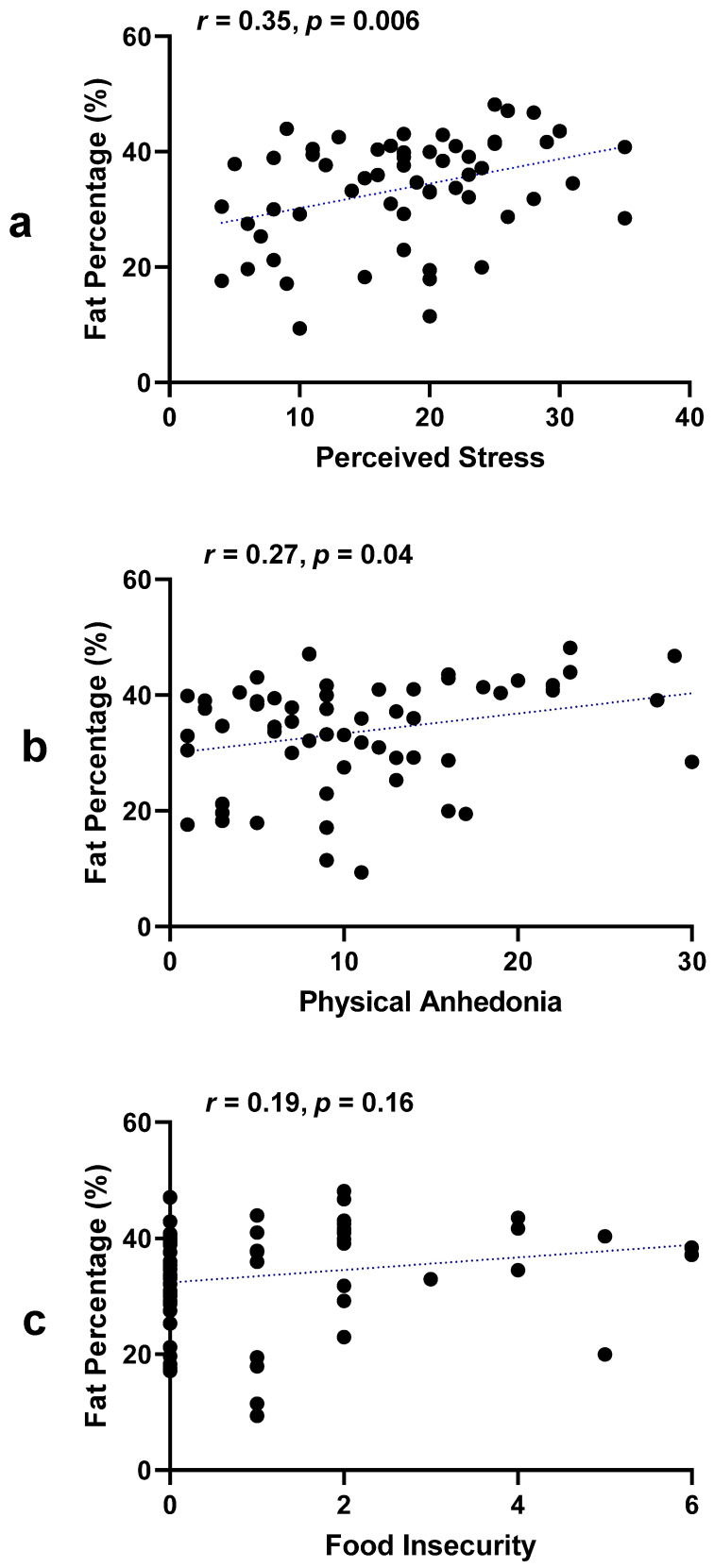
Associations of percent fat with stress, anhedonia, and food insecurity. (**a**) Pearson correlation coefficient between perceived stress and fat percentage [*r* = 0.35, *p* = 0.006]; (**b**) Pearson correlation coefficient between physical anhedonia and fat percentage [*r* = 0.27, *p* = 0.04]; (**c**) Pearson correlation coefficient between food insecurity and fat percentage [*r* = 0.19, *p* = 0.16].

Greater perceived stress and anhedonia scores were correlated with higher percent body fat (*r* = 0.35, *p* = 0.006; *r* = 0.27, *p* = 0.04, respectively), but food insecurity was not (Figure 1). Subjective social status was not associated with perceived stress, anhedonia, or food insecurity.

**Figure 2:**
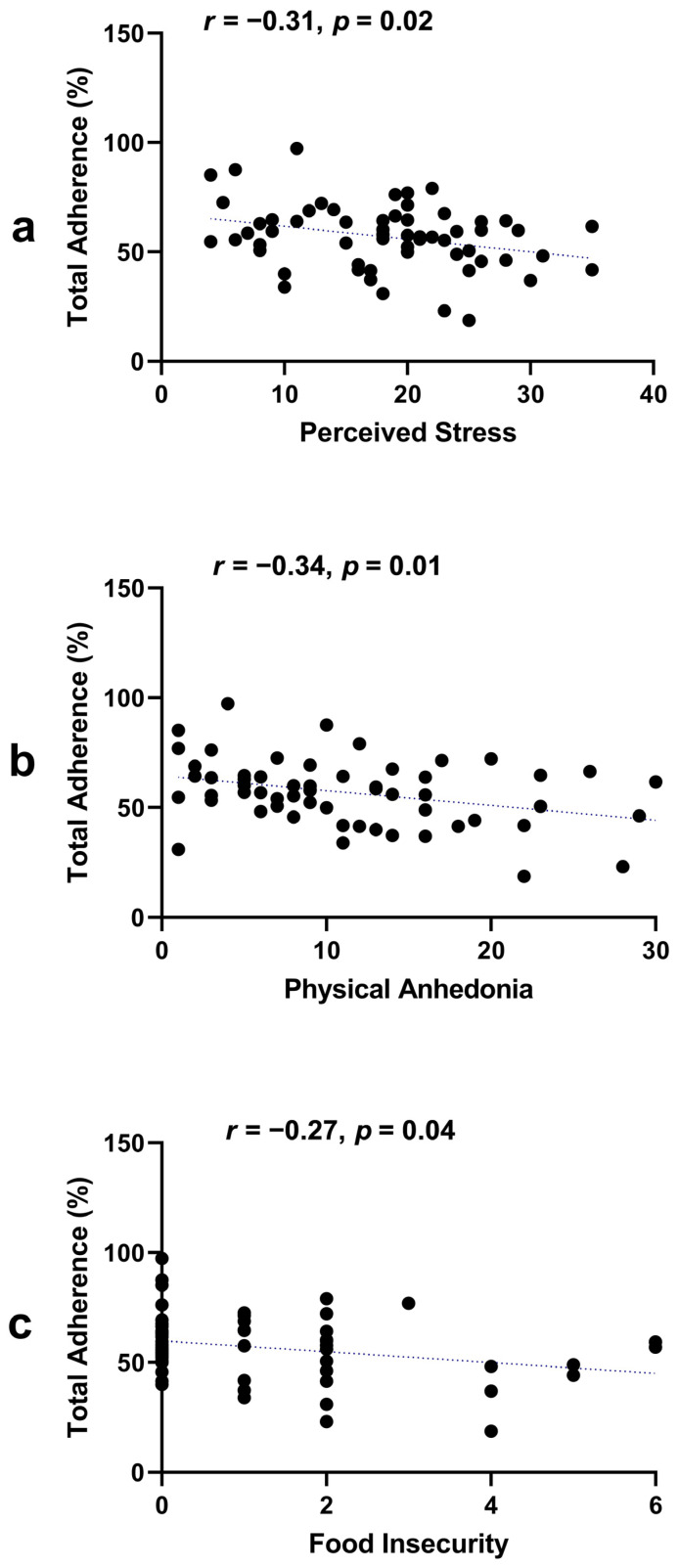
Associations of total adherence with stress, anhedonia, and food insecurity. (**a**) Pearson correlation coefficient between perceived stress and total adherence [*r* = −0.31, *p* = 0.02]; (**b**) Pearson correlation coefficient between physical anhedonia and total adherence [*r* = −0.34, *p* = 0.01]; (**c**) Pearson correlation coefficient between food insecurity and total adherence [*r* = −0.27, *p* = 0.04].

Perceived stress (*r* = −0.31, *p* = 0.02), anhedonia (*r* = −0.34, *p* = 0.01), and food insecurity (*r* = −0.27, *p* = 0.04) were associated with total 6-week percent adherence scores, but subjective social status was not (Figure 2). Refer to Supplemental Table S1 for a complete correlation matrix.

### 3.2. General Linear Model Analyses

In all adjusted models, percent body fat was not associated with adherence. In general linear models, higher perceived stress, anhedonia, and food insecurity scores remained associated with decreased adherence (*B* = −0.72, *p* = 0.005, CI = −1.21, −0.22; *B* = −0.68, *p* = 0.01, CI= −1.20, −0.16; *B*= −2,39, *p* = 0.04, CI= −4.70, −0.07, respectively). These associations remained for perceived stress and anhedonia after the inclusion of subjective social status, but not food insecurity (Table 2). Food insecurity parameter estimates, although no longer significant following the inclusion of subjective social status (Model 1: *p* = 0.043; Model 2: *p* = 0.063), did not change.

## 4. Discussion

In this secondary analysis of a randomized clinical trial, higher levels of perceived stress, anhedonia, and food insecurity were significantly associated with lower adherence. These associations remained significant for perceived stress and physical anhedonia even after controlling for age, sex, percent body fat, and subjective social status.

Psychosocial factors (e.g., stigma, perceived stress) strongly influence daily eating behaviors and self-regulation processes [24]. Specifically, food insecurity, socioeconomic status, and overall ratings of mood alter adherence to both medical and dietary recommendations [11,43,44]. Our findings of decreased adherence to a dietary intervention in individuals reporting greater food insecurity and higher levels of perceived stress and anhedonia are consistent with the prior literature and theory [9,13,14,15]. Most importantly, food insecurity, anhedonia, and perceived stress are defined psychosocial measures that predicted lower adherence even after accounting for body fat.

Anhedonia has been associated with maladaptive eating behaviors and less successful weight loss [17,45,46,47]. During a weight loss intervention study, participants with anhedonia had higher binge eating, emotional eating, and uncontrolled eating rates and a lower BMI decrease than those without anhedonia [47]. We previously observed that greater anhedonia in individuals with obesity mediated poorer decision-making task performance, linked to deficits in reward processes such as motivation and pleasure [48,49,50]. Prefrontal cortex areas are responsible for executive decision making; thus, poorer scores on decision-making tasks may reflect altered prefrontal activity. Indeed, a lower prefrontal volume is associated with reduced exercise adherence. Higher anhedonia is associated with reduced connectivity between nucleus accumbens and prefrontal cortex areas in individuals with major depressive disorder [51]. Projections between the nucleus accumbens and the ventral tegmental area are considered a central pathway for the reward circuitry, including the recognition and consumption of rewards in the environment [52]. Thus, anhedonia may modify decision making that affects adherence via a reduction in the activation of this brain pathway, and could be one explanation for our observed association between increased anhedonia and decreased adherence.

Our findings of an association between greater perceived stress and lower adherence are consistent with past research demonstrating a lower observance of dietary guidelines in patients with Type I diabetes [13] as well as diminished adherence with HIV antiviral [53] and hypertension medicines [54] in participants with higher levels of perceived stress. Previous research from our unit showed that perceived stress is associated with short-term weight gain [22], suggesting that perceived stress may have a concurrent effect on health-maintaining behaviors such as healthy eating [55,56] and weight loss [57], both directly and through behavioral and physiological intermediaries [24]. Exposure to stress is related to reduced activity In brain regions that affect adherence, including the prefrontal cortex, amygdala, and hippocampus [58]. Lower adherence is related to lower activity or reduced regional gray matter volume in similar brain regions such as the prefrontal, motor, somatosensory, temporal, and parietal regions [59,60]. Thus, exposure to stress may affect adherence via the modulation of brain regions and pathways.

Subjective social status was not associated with perceived stress, physical anhedonia, or food insecurity; however, the relationship between food insecurity and dietary adherence was no longer statistically significant when social status was held constant [61]. Currently, the way in which perceived social status might modify this relationship is not well understood. Individuals experiencing higher levels of food insecurity tend to exhibit increased rates of carbohydrate oxidation and decreased rates of lipid oxidation [61]. These metabolic factors are known contributors to overeating and weight gain [62,63,64,65], which could potentially elucidate the connection between greater food insecurity and decreased adherence to dietary guidelines. The fact that this relationship is influenced by subjective social status suggests that improvements in perceived social status may possess sufficient influence to counteract metabolic drivers. Given the significant impact of subjective social status on health outcomes [58], often above and beyond objective measures of socioeconomic status [40,66,67], interventions aimed at enhancing these perceptions could serve as an additional strategy for combating weight gain.

Our study had several strengths, including the use of multi-rater, multiple assessments of adherence as well as the inclusion of validated measures to assess perceived stress, anhedonia, and food insecurity, which have not been previously well studied in association with dietary adherence. Also, all meals were provided by our metabolic kitchen, thus eliminating the burden of participants having to prepare their own daily meals. Our analysis also had limitations. First, a relatively small sample size may have limited statistical power to examine associations within the calorie-reduced dietary group. Second, the mean age of participants was 53 years old and thus generalizability of the findings may be limited to older individuals; however, this could also be considered a strength given that much of the literature on psychosocial associations with eating behavior is in college-age participants. Third, while we consider providing food to participants as a strength, it may potentially limit generalizability given that most free-living individuals need to prepare their own meals. Fourth, the intervention was relatively short term and further studies are needed to understand the impact of psychosocial variables on long-term dietary adherence.

## 5. Conclusions

Our findings demonstrate that perceived stress, anhedonia, and food insecurity play more important roles than weight status and should be prioritized as targets for improving adherence in dietary and even other medical interventions.

## Figures and Tables

**Table 1:** Demographics of study population (unadjusted).

	Total (n = 60)	Normal Weight (n = 23)	Individuals with Obesity (n = 37)	*p*
Age (years)	53.3 (14.5)	54.8 (12.8)	52.4 (15.5)	0.55
Sex				0.92
Males	23	9	14	
Females	37	14	23	
Race/Ethnicity				**<0.001**
Indigenous American	15	1	14	
White	31	18	13	
Hispanic	7	1	6	
Black	3	1	2	
Asian	2	2	0	
Other	2	0	2	
Subjective Social Status (Community level)	6.9 (2.1)	6.9 (2.1)	6.8 (2.1)	0.83
Height (cm)	166.6 (9.9)	167.1(8.6)	166.3 (10.7)	0.76
Weight (kg)	86.3 (27.4)	63.7 (8.2)	99.8 (25.8)	**<0.0001**
Body Mass Index (kg/m^2^)	30.9 (8.6)	22.7 (1.8)	35.8 (7.2)	**<0.0001**
Body Fat (%)	33.7 (9.4)	25.3 (8.7)	38.7 (5.5)	**<0.0001**
Fat Mass (kg)	30.3 (15.5)	16.0 (5.6)	38.8 (13.1)	**<0.0001**
Fat-Free Mass (kg)	56.0 (14.8)	47.6 (9.1)	61.0 (15.3)	**0.0005**
Perceived Stress	18.2 (7.7)	15.4 (7.8)	19.9 (7.3)	**0.0258**
Anhedonia	11.2 (7.5)	9.6 (7.3)	12.1 (7.5)	0.20
Food Insecurity	1.2 (1.6)	0.83 (1.2)	1.4 (1.8)	0.20
Adherence (%)	57 (15)	59 (10)	56 (17)	0.42

Note: Bolded *p*-values (*p* < 0.05) denote statistically significant differences between the two groups (normal weight vs. obesity).

**Table 2:** General linear model analysis: Adherence (%) with psychosocial variables.

	Model 1				Model 2			
	B	*p*	95% CI		B	*p*	95% CI	
**Perceived Stress**	−0.72	**0.005**	−1.21	−0.22	−0.76	**0.004**	−1.27	−0.25
**Anhedonia**	−0.68	**0.01**	−1.20	−0.16	−0.70	**0.01**	−1.23	−0.17
**Food Insecurity**	−2.39	**0.04**	−4.70	−0.07	−2.38	0.06	−4.89	0.14

**Model 1** covariates: age, sex, percent body fat. **Model 2** covariates: age, sex, percent body fat, subjective socioeconomic status (community level). Bolded *p*-values denote statistical significance (*p* < 0.05). Note: Perceived stress, anhedonia, and food insecurity were in separate multivariable models, along with covariates.

## Data Availability

Data described in the article will be made available upon request pending application and approval by the Institutional Review Board of the NIDDK.

## Supplementary Materials

The following supporting information can be downloaded at: https://www.mdpi.com/article/10.3390/nu16040526/s1, Figure S1: CONSORT diagram; Table S1: Correlation Matrix.
