# Supplementary material for: Dietary Adherence Is Associated with Perceived Stress, Anhedonia, and Food Insecurity Independent of Adiposity
Source: Nutrients. 2024 Feb 14;16(4):526. doi: 10.3390/nu16040526 (PMC10892668; doi:10.3390/nu16040526)
Supplement: Supplementary file 1 [file nutrients-16-00526-s001.zip › Supplemental Table S1.pdf]

Supplemental Table S1: *Correlation Matrix*

	**1**	**2**	**3**	**4**	**5**	**6**	**7**	**8**
1. Age	--	0.02	-0.17	0.04	0.11	-0.15	-0.09	0.25
2. Sex (M = 1; F = 2)	0.01	--	**0.45**	0.02	0.03	0.01	-0.06	0.07
3. Body Fat (%)	-0.11	**0.49**	--	-0.14	**0.37**	**0.26**	**0.27**	-0.13
4. Subjective Social Status	0.11	0.01	-0.15	--	**-0.29**	-0.22	-0.13	-0.08
5. Perceived Stress	0.08	0.05	**0.35**	-0.23	--	**0.42**	**0.37**	-0.25
6. Anhedonia	-0.21	0.06	**0.27**	-0.19	**0.46**	--	**0.26**	**-0.29**
7. Food Insecurity	0.00	-0.05	0.19	-0.19	**0.36**	0.19	--	-0.22
8. Total Adherence	**0.33**	0.03	-0.09	-0.04	**-0.31**	**-0.34**	**-0.27**	--

Bolded coefficients denote statistical significance ($p < 0.05$)

Note: Pearson correlation coefficients (*r*) are below the diagonal and Spearman correlation (*ρ*) coefficients are above the diagonal.
