# Supplementary figures and images for: Dietary Adherence Is Associated with Perceived Stress, Anhedonia, and Food Insecurity Independent of Adiposity
Source: Nutrients. 2024 Feb 14;16(4):526. doi: 10.3390/nu16040526 (PMC10892668; doi:10.3390/nu16040526)

**Supplemental Figure S1.** CONSORT diagram.

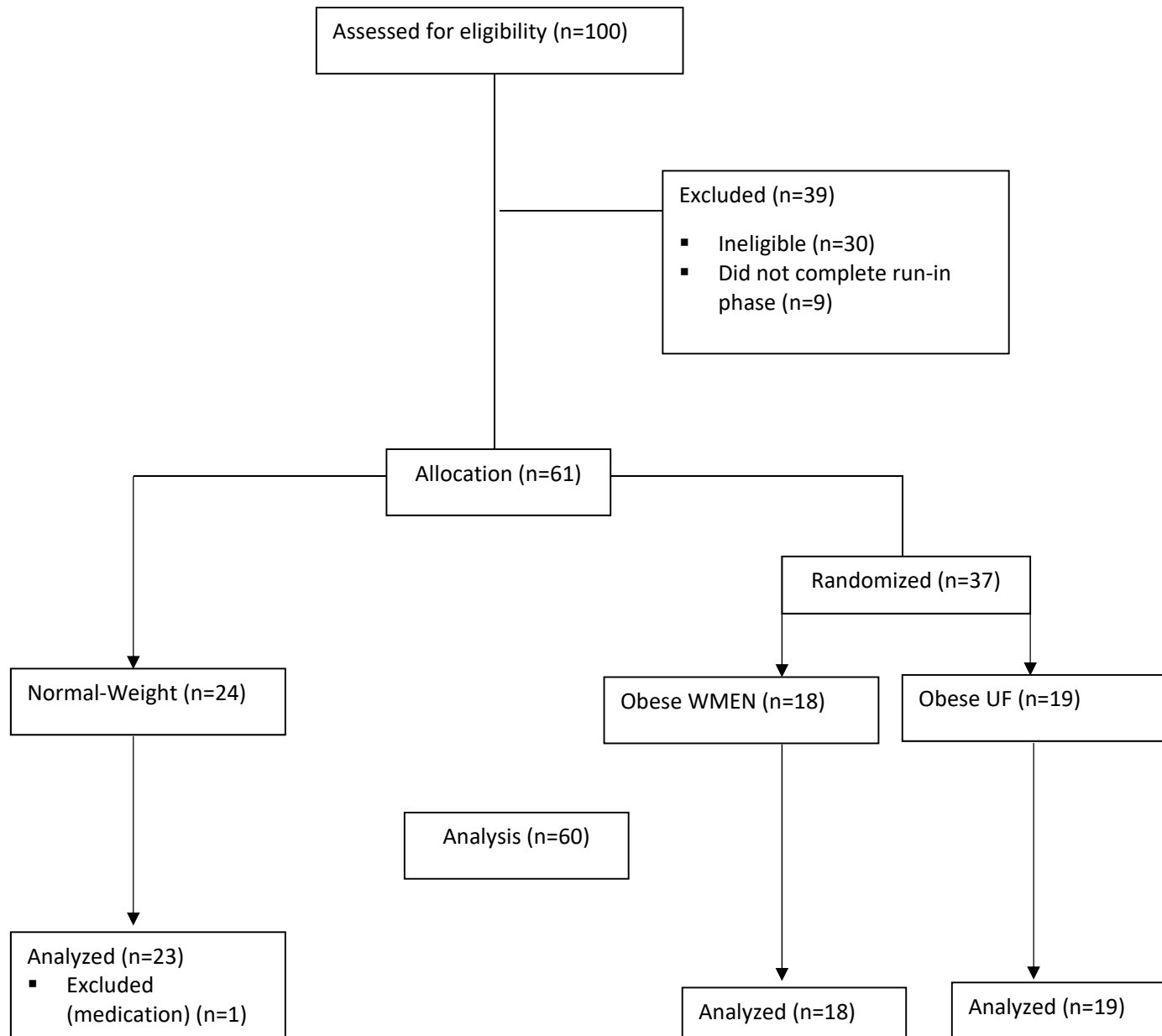

Supplement: Supplementary file 1 [file nutrients-16-00526-s001.zip › Supplemental Figure S1.pdf]
